# Error evaluation in the laboratory testing process and laboratory information systems

**DOI:** 10.5937/jomb0-31382

**Published:** 2022-02-02

**Authors:** Azila Arifin, Maryati Mohd.-Yusof

**Affiliations:** 1 University Kebangsaan Malaysia, Faculty of Information Science and Technology, Bangi, Selangor, Malaysia

**Keywords:** case study, error, evaluation, framework, laboratory information systems, Lean, patient safety, total testing process, socio-technical, proučavanje slučaja, graška, procena, laboratorijski informacioni sistem, LEAN, sigurnost pacijenta, ukupni proces ispitivanja, socio-tehnološki proces

## Abstract

**Background:**

The laboratory testing process consist of five analysis phases featuring the total testing process framework. Activities in laboratory process, including those of testing are error-prone and affect the use of laboratory information systems. This study seeks to identify error factors related to system use and the first and last phases of the laboratory testing process using a proposed framework known as total testing process-laboratory information systems.

**Methods:**

We conducted a qualitative case study evaluation in two private hospitals and a medical laboratory. We collected data using interviews, observations, and document analysis methods involving physicians, nurses, an information technology officer, and the laboratory staff. We employed the proposed framework and Lean problem solving tools namely Value Stream Mapping and A3 for data analysis.

**Results:**

Errors in laboratory information systems and the laboratory testing process were attributed to failure to fulfill user requirements, poor cooperation between the information technology unit and laboratory, inconsistency of software design in system integration, errors during inter-system data transmission, and lack of motivation in system use. The error factors are related to system development elements, namely, latent failures that considerably affected the information quality and system use. Errors in system development were also attributed to poor service quality.

**Conclusions:**

Complex laboratory testing process and laboratory information systems require rigorous evaluation in minimizing errors and ensuring patient safety. The proposed framework and Lean approach are applicable for evaluating the laboratory testing process and laboratory information systems in a rigorous, comprehensive, and structured manner.

## Abstract (sr)

**Uvod:**

Proces laboratorijskog ispitivanja sastoji se iz pet analitičkih faza u ukupnom procesu analiziranja. Aktivnosti u laboratorijskom procesu uključuju i one koje se odnose na utvrđivanje grešaka i utiču na laboratorijski informacioni sistem. Ovo izučavanje ima za cilj identifikaciju grešaka u odnosu na primenu sistema od prve do poslednje faze ispitivanja u laboratorijskom procesu primenom poznatog informacionog sistema za celokupni proces ispitivanja.

**Metode:**

Primenili smo kvalitativno izučavanje procesa u dve privatne bolnice i medicinske laboratorije. Podatke smo sakupljali putem intervijua, na osnovu posmatranja i dokumentovanih metoda analiziranja uključuju i lekare, sestre, informacionog stručnjaka i laboratorijsko osoblje. Primenili smo poznati LEAN proces za rešavanje problema koji je poznat kao Value Stream Mapping i A3 za analizu podataka.

**Rezultati:**

Greške u laboratorijskom informacionom sistemu i laboratorijskom procesu ispitivanja javljaju se uglavnom zbog loše saradnje između jedinice za informacione tehnologije i laboratorije, zbog lošeg informacionog sistema, prenosa podataka i motivacije za primenu sistema. Greške nastaju i zbog problema u razvoju samog kvaliteta informacionog sistema. Takođe greške su posledica lošeg servisiranja sistema.

**Zaključak:**

Kompleksan laboratorijski proces ispitivanja i laboratorijski informacioni sistem iziskuju rigoroznu procenu i kontrolu grašaka i osgiranje sigurnosti pacijenata. Predloženi okvir i primena LEAN postupka su neohodni za procenu procesa laboratorijskog ispitivanja i laboratorijskog informacionog sistema koji moraju da budu rigorozni i sveobuvatni.

## Introduction

A mistake or inefficiency in one of the stages of the laboratory testing chain can affect the overall process implementation and management, and subsequently physician diagnosis [1] [2]. A laboratory information systems (LIS) expedites and facilitates interactions during the laboratory testing process [3]. Involvement of multiple units in testing workflow requires effective use of LIS to monitor task performance, ensure a smooth process, and readily identify errors. Many errors identified in laboratory test results were caused by a complex, error prone, unreliable, and poorly designed LIS [4] [5]. These outcomes are aggravated when the LIS linked patient and test data to other units and institutions and involved data exchange because of complex inter system interaction [6]. Errors were also attributed to human factors, including patient misidentification and an erroneous test request [7].

**Figure 1:**
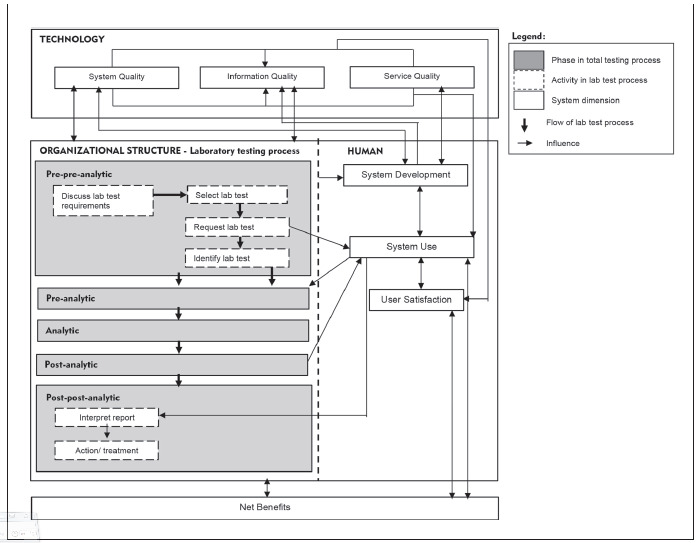
The proposed TTP-LIS framework

Total testing process (TTP) [8] is a unique framework that guides the testing process as well as analyzing and minimizing testing error risk not only in the laboratory center, but also in other clinical units [7] [9]. The TTP includes internal and external laboratory activities that involve one or more procedures requiring staff interaction. We proposed a TTP-LIS posed framework on the basis of a combination of TTP and human, organization, technology and fit (HOT-fit) frameworks [10] [11]. The HOT aspects are crucial elements that complement the evaluation of the LIS and lab testing process. The proposed framework aims to illustrate a systematic, coordinated, and optimized laboratory testing process and LIS flow to facilitate a rigorous error evaluation [12]. The evaluation factors, dimensions, measures and their relationships are depicted in Figure 1.

Error evaluation can benefit from Lean, a quality improvement method that emphasizes on removing process waste, including error. Various Lean tools, such as value stream mapping (VSM), 5Why, and A3 problem-solving methods, have been widely used for process improvement [13]. A3 is a structured approach to problem solving that uses a report tool to summarize the definition, scope, discovery process, findings, proposed action steps, and results from the problem analysis. A3 can be combined with other Lean tools, such as VSM and 5Why, to visualize and identify the root cause of problems. VSM is used to illustrate the overall process to identify waste/problems and the appropriate solutions in the current and future state map, respectively. The problem can be scrutinized using the 5Why tool to identify its root cause and mitigation strategy by asking a series of question, either five times or any appropriate range. The study focused only on pre-pre-analytic and postpost-analytic phases of the TTP framework, given their high error rates [14] [15], compared to other phases.

## Material and Methods

We conducted a subjectivist case study strategy employing qualitative methods in this summative evaluation to examine errors related to the LIS and the first and last phases of the lab testing process. A subjectivist approach enabled a comprehensive understanding of the healthcare context surrounding the management of LIS-induced error by generating detailed, insightful explanations [16] [17]. We performed evaluation by applying the TTP-LIS framework at two premier private hospitals in Malaysia. These cutting-edge hospitals have been leading the national health care and are recognized by accreditation bodies such as the Malaysian Society for Quality in Health, Joint Commission International (XI), and Quality Management System (MS ISO 9001: 2015). The local Institutional Review Board deemed this study exempt from review. Author AA, a trained qualitative researcher, collected the data through interviews, non-participant observations, and document/artifact analysis methods.

### Sampling

A purposeful snowball sampling method provided in-depth information from key informants. We identified participants from our initial contact with the lab director. We discussed the appropriateness of selected informants with the lab head based on their respective expertise, job scope and abilities in providing the required information. Finally, we recruited 15 participants, including clinicians and management, lab, and IT staff (Table 1).

**Table 1:** Participant list and method description

Method	Participant (N)	Description
Interview	Physician (2)<br>Nurse (2)<br>Lab head (1)<br> Lab staff (2)<br>IT staff (1)	• Semi structured interview questions were formulated according to the job description and role of participants
	**Total =** **8**	
Document analysis	Physician (1)<br>Lab head (1)<br>Lab technician (1)<br>IT staff (1)	• Lab test request form<br>• Statistical report of the lab test request form<br>• Statistical report of the lab test results (non/late access, location, and test type)<br>• Monthly/annual report<br>• Improvement in the lab testing process<br>• LIS improvement report (based on modules/ functions/others)
Observation	Lab head (1)<br>Lab staff (2)	• Process flow of the lab test request<br>• Lab test report process
	**Total = ** **15**	

### Data collection and analysis methods

The face-to-face, one-on-one interview lasted for one to two hours for each informant who we queried on lab testing process, LIS use, error and mistake incidents, their causes, and the strategies for mitigation and LIS improvement. We audio recorded and transcribed interviews. Observation took place in a medical lab for over a day on lab testing processes, from clinical requests to the production of lab results, to identify potential LIS-induced errors. We analyzed documents related to LIS' overall development, operation and management, process owner, backup system handling, and software and hardware management. We analyzed data thematically using the initial TTP-LIS evaluation framework [12]. In addition, we employed three Lean tools, namely VSM, A3 *Problem Solving*, and 5Why to visualize the current process, its problems and root cause, and the desired (future) state of the first and last phases of lab testing [13]. We validated and refined the TTP framework with an expert who reviewed and acknowledged the said framework as a comprehensive evaluation tool for the lab testing process and LIS.

## Results

**Figure 2:**
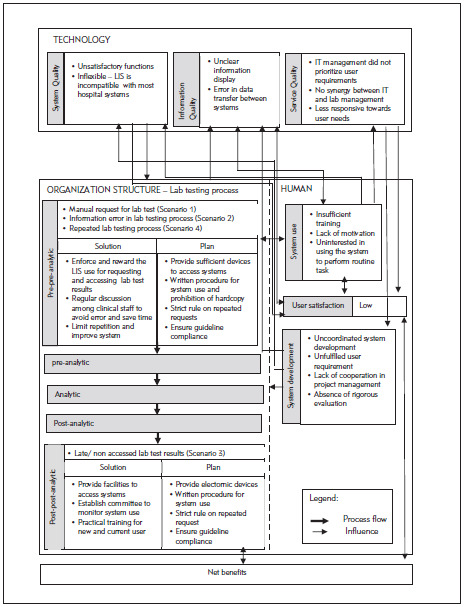
Error factors in the lab testing process and LIS

The hospitals PHA and PHB were established in the mid-1990s. They collaborated with a private laboratory, Lab C, which has managed most lab operations at all PH branches since 2000. The hospitals provide services to 3000 to 4000 patients at a time and provide educational services to medical and nursing students. Evaluation of the overall system used in the hospitals and laboratories involved the LIS, lab testing process and other health information systems (HISs). The LIS evolved from a stand-alone system that only supports internal laboratory operations to a system with extended functions that are connected to HISs. The LIS was also developed by the IT unit of Lab C whereas the HIS was outsourced and operated by the hospital IT unit. Both systems are integrated in a new platform. The IT staff in Lab C provide training to LIS users Figure 2. illustrates the overall findings according to the proposed TTP-LIS framework.

### Human factors

Overall, the LIS was optimized by the lab staff compared to the hospital staff. Many clinicians did not attend training because of time constraints and their heavy workload. Lack of training and exposure to LIS result in low system use. Users, particularly senior physicians and nurses, are reluctant to use the LIS to request lab tests and access its results for various reasons such as »wasting time, hassles to remember password, patient name or id« (Lab Head). According to a physician, »system use disrupted my task. Sometimes the LIS processes data slowly and requires time consuming access, while the network is disrupted during lab test request. The manual form saved more time.« A nurse stated that although »system use eased our task, our competency is low«.

LIS use is mandatory only in some PH branches, while others still operated manually. The LIS use started from the laboratory and expanded to clinical units. However, poor synergy and discrepancies between management and IT in planning and strategizing the LIS affect system development and the subsequent non-optimized LIS use in clinical units. Poor system development is also attributed to poor service quality in terms of responsiveness, assurance (service providers' skills, consideration and ability to provide trust and confidence [18]), and empathy from the service provider and hospital management. Decisions for system development were made according to individual or other interest including politics, such as conflict of interest and business profit, instead of system use. The integration of heterogeneous, outsourced, and in-house developed systems with different platforms, hardware, and software resulted in many system problems, such as unreadable information, unclear images (blurred, inappropriate pixel sizes, and display of system coding), and inaccessible information. These problems pose challenges to the clinical unit and the physicians' decision making pertinent to patient diagnosis or treatment because of inaccurate data. Subsequently, these issues affect system use, user satisfaction, and the lab test process. Physicians and nurses preferred the manual method in requesting lab tests and obtaining lab test results as they perceived as faster than those of LIS. Instead of increasing process efficiency, LIS use delayed tasks and disrupted the decision-making process. In short, system development outcomes significantly affect the system and information quality, and service quality determines the fulfillment of user requirements.

### Technology factors

System quality influenced other factors including system development, system use, the lab testing process, and user satisfaction. We identified errors that stemmed from poor LIS functions, including the number of lab test results that are less than the actual number of applied tests. Moreover »[some] lab test results accessed from LIS showed unexpected analysis when the results are linked to diagnosis results from the CIS« (Dr. B).

### Organizational factors

The whole lab testing process takes around 15-20 minutes, if there is no disruption, to paste bar code on specimen tubes and application form, entering request information in LIS, testing specimen and verifying lab test results. We chose to analyze four process scenarios that were recommended by the informants according to their error impact on the overall workflow in terms of additional time, increased workload, material waste, and (most importantly) delay in patient treatment. Scenario 1 (manual request of the lab test process and printing lab testresults) became problematic as it resulted in extra workload for lab staff to routinely check or request missing information on the manual form, file, print documents, and »...the patient code on manual forms need to be individually scanned and checked to ensure its consistency with the system« (lab head). Then, the lab test results must be printed and sent tophysicians or nurses. Missing or lost results required another print out and the same goes for physicians who request patient lab test histories. Increased burden arises from the error chain, whereas a physician’s error rippled to the lab unit and the prescribing process that involves lab test results.

Erroneous test request (Scenario 2) occurred due to several reasons, as claimed by the informants. »We must perform the test upon receiving the sample and request form. We would not able to identify the request as a mistake when the request information is consistent with those of the system« (lab staff). »Choosing the wrong test commonly happened in critical situations where [the] physician does not have time to check [the] test requested by the nurse« and the nurse »forgets to verify it with the physician.« A mistake is usually realized upon test completion. Non accessed/delayed lab test results (Scenario 3) recurred because of non-scrutinized processes or hasty decisions. According to the lab head, the situation affects staff efficiency, particularly when they must prioritize other urgent lab tests. Lab staff were puzzled when »a requested test results were not accessed upon its completion, [thereby] indicating that the test is not needed, [a situation] which wasted our time and resources to conduct the test.«

In Scenario 4, the repeated lab testing process is attributable to the inefficiency of the clinical unit and sample testing process. Lab testing is repeated when the laboratory or physician identified test results that are abnormal or fall outside the reference range lab test or unidentified errors were present in the test request. Upon realizing these abnormalities and erroneous request during results validation, the lab head ordered a second and correct test request, respectively. If the first and second test results are consistent, they are categorized as a critical case and the physician is contacted immediately. Result abnormalities are entailed for the second test, whereas erroneous request attributable to staff carelessness or inefficiency should be avoided. Similar to Scenario 2, the prescriber's verification is imperative before submitting the test request.

According to the four scenarios of the two lab testing processes for pre-pre-analysis and post-post-analysis (Figure 1), A3 diagrams are used to illustrate and elaborate upon the as-is and to-be processed elements as demonstrated in Scenario 2 (Figure 2). The process is related to lab test request by a nurse or clinical assistant using the LIS. A nurse was instructed by a physician to request for a lab test using a CIS. The nurse labelled sample tubes and stored them while waiting for a lab staff member to collect them. Then, the nurse directly entered the related information for requesting the lab test in the computer unit. However, the test type that she chose differed from that desired by the physician.

Normally, neither the nurse nor the lab head would realize the mistake until the physician checks the order before submitting it to the LIS. Therefore, the test was processed normally according to the requested test type. Upon the test completion, the results were generated, checked, and verified by lab head. Then, the results were submitted to the CIS via the LIS. A physician accessed the lab test results, only to realize that they are irrelevant. At this point, the charge was already forwarded to the finance unit for patient billing. This mistake required the physician to report the occurrence to the management and finance, and the charge must be paid by the hospital. Therefore, double checking and verifying test requests are critical to avoid a chain of problems. The physician is responsible for rechecking requests, and the nurse must remind the physician about it before submission. We illustrated the problems to aid in identifying the root cause and planning for mitigation as follows.

### A3 Problem Solving report for Scenario 2

#### Issue

Mistake in selecting lab test type during the request through the LIS.

#### Background

The nurse received instruction from the physician to request for a lab test via the LIS. The nurse did not realize that she had mistakenly chose the wrong test type during the request process.

#### Future State

**Figure 3:**
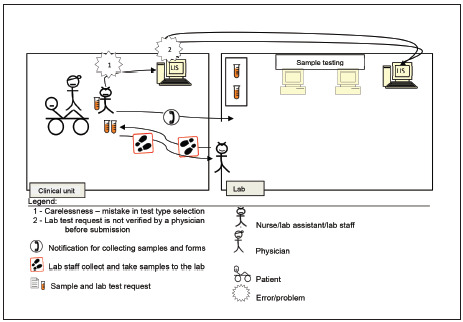
Current state<br>As-is Process of Scenario 2

The to-be processed flow diagram is similar with that of the as-is process (Figure 3), except for the replacement of the two problems with the following two solutions.

#### Solution Steps

Detailed discussion among the medical team of a mitigation plan to avoid recurring mistakes and resources waste. (Table 2) (Table 3) (Table 4)

**Table 2:** Problem analysis

1. Carelessness – mistake in test type selection	
Why?	Lack of focus in receiving order
Why?	Irrelevant text/unclear test type selection/unfriendly user interface resulting in user misunderstanding
Why?	Poor system/ interface design
2. Lab test request is not verified by a physician before submission	
Why?	Slip due to forgetfulness
Why?	Urgency to submit the test request
Why?	Non-compliance with the standard operating procedure (SOP)

**Table 3:** Implementation plan

Plan	Expected results
Lab test verification/auto verification is included as part of SOP in using LIS	The procedure for lab test verification is followed
Impose the procedure (e.g.: reminder on main web/mobile LIS interface, awareness campaign, training, and poster)	Physicians and staff are aware of and adhere to the procedure
	

**Table 4:** Costs/benefits

**Cost**
System upgrade to include auto verification and alert functions
Awareness intervention programs
**Benefits**
Reduced mistakes in lab test requests
Increased efficiency and reduced turnaround time for lab test request and initializing patient treatment

## Discussion

We went through a relatively challenging, iterative process of constructing structured and comprehensive socio-technical factors in the TTP-LIS framework [12]. This study contributes to the existing knowledge by proposing a new framework based on the HOT-fit and TTP frameworks, as well as concepts in error management and process improvement, namely the Lean methods. The TTP-LIS framework features a comprehensive evaluation method of sociotechnical factors that can be applied flexibly in other processes and systems in a similar or different clinical settings. The findings showed the practicality of the TTP-LIS framework as an evaluation tool in identifying errors and their causal factors. The use of Lean tools, namely, A3 report, VSM, and 5Why, enabled us to analyze and visualize the root cause of problems in an objective and structured manner [13] [19]. The evaluation of LIS-induced error enabled the IT staff in both laboratory and hospital to collaborate in improving LIS quality by synchronizing system development to reduce system integration problems and considering system functions according to user requirements. Human, lab testing process, organization and technology factors are intertwined. Errors caused by human [4] [7] technology [5], and processes [3] [9] disrupted the lab testing process workflow. Human factors mainly contribute to errors in the lab testing process and LIS, as proven in other studies [7]. Errors in system development and use that are attributed to human factors require continuous evaluation and monitoring to ensure quality. The LIS supports user needs [3] [20] and routine tasks and reduces problems [21]. Mandatory use of the LIS among physicians and nurses is meant to increase the efficiency of routine tasks in the lab testing process. However, LIS use among clinicians is very low. In general, the findings can be categorized as follows: latent failure in system development, poor error management, and unsatisfactory lab testing process and LIS use.

### Latent failure in system development

System development highly contributed to error occurrence in the LIS and HIS use in terms of introduction of new technology, heterogeneous software, human-computer interaction, and communication issues within the system developer team. These factors are consistent with other findings [3] [5] [6]. These latent failures hinder the optimized potentials of the LIS. The case LIS developers really understand the requirements of the lab testing process and featured them as the main functions in LIS. In contrast, the HIS was outsourced; the hospital management team identified more general user requirements. This resulted in integration conflict and subsequent errors, including unclear data requirement and inappropriate graph generation that that affect physician decision making.

Latent failure is a major challenge for management and organizational decision makers. Strong collaboration between management with both hospital and laboratory units can aid in solving latent failure [22]. During the system development, risk factors should also be considered apart from the cost. Heterogeneous system development methods increased error risk and cost. On the contrary, a unified system development method that considered user requirement reduced error risk. The study can be extended to further understand latent failure factors and identify optimum strategies to address them.

### Poor error management

In general, LIS-induced errors require tackling the problems at their root cause and employing a basic solution method from the socio-technical perspectives, before quality improvement and automation [3] [23] [24] [25] [26], as proposed in our error management approach. Most identified errors can be mitigated through a joint, multi discipline collaboration from all staff. However, monitoring is imperative at the outset [27] to ensure guideline compliance. An error management method serves as a tool to mitigate errors identified by the system or through routine error checking at the end of a task completion. The absence of an error management system led to recurring errors [28] [29] that waste time, resources, and cost in terms of service or materials. Recurring errors also indicate ineffective and inefficient workflow and system use that negatively affects work motivation. Many error management strategies have been successfully proven in other industries and can be adopted in laboratory and clinical settings. These strategies include 1) reducing cognitive load through automated record, notes, and process (e.g., verification and checking); 2) enhanced information access; 3) imposing an error-proofing function for critical tasks such as preventing fatal drug instruction according to the dosage for certain patients; 4) checking error at its source (individual process step); 5) coordination of similar tasks; and 6) minimizing individual involvement in a single task [30] [31] [32] [19].

### Lab testing process and LIS use

User acceptance and sufficient training increase LIS use in lab testing workflow and subsequently ensure smooth flow and enhanced work quality [3] [7] [21] [33]. However, a lean workflow is imperative prior to optimizing the process automation to improve the core issues in the workflow itself [3] [13]. Various efforts have been made to reduce errors in routine monitoring, particularly in the early and final phases of the lab testing process, given that both phases involve clinical instead of lab staff who are more familiar with the related process. Therefore, inter departmental cooperation is crucial for avoiding recurring errors.

In short, although all scenarios involved simple errors and mistakes, they posed various possible implications, such as inefficiency, high workload, adverse events, and patient safety issues. Inappropriate testing is not only wasteful and costly, but also risky to patients [32]. However, the processes can be streamlined and optimized through management and mitigation of process and error. Automated interventions such as an ordering system that alerts prescribers can educate them about requesting inappropriate or repeated testing [32]. Moreover, auto verification is widely reported to have potential for facilitating safe, efficient, and reliable tools [31] [34]. We proposed a comprehensive plan to avoid errors in the early and final lab testing process. The steps include

- analyzing and redesigning workflow according to Lean methods;
- establishing clear, written, and digital procedures;
- improving system training for users;
- outlining indicators for quality monitoring;and
- improving communication and synergy amonghealthcare and laboratory professionals.

The procedure for lab testing workflow must clarify patient identification; gathering, labelling, and transferring specimens; and analysis preparation. The responsible individual must understand and acknowledge the procedure and its importance, the potential risk, and effect on the sample and subsequently to the patient because of procedure noncompliance. All steps required ongoing training and efficient assessment.

## Dodatak

### Study limitations

The short duration of the observation limited the detail evaluation of possible error incident during the lab test process but this situation was offset with a briefing from the lab head. Moreover, documents related to LIS use and the lab testing process are restricted as they are regarded as private and confidential. Furthermore, manual requests for laboratory tests limit the evaluation of LIS use in clinical units, particularly in the pre-pre-analysis phase. Nevertheless, the rich interview data compensate for this constraint.

### Author contributions: 

All authors accept responsibility for the entire content of this manuscript and have approved its submission.

### Informed consent: 

We obtained informed consent from all individuals involved in this study.

### Acknowledgements: 

This work was supported by the Ministry of Higher Learning Malaysia (Grant no.s FRGS/1/2018/ICT04/UKM/02/5 and ERGS/1/2011/STG/UKM/02/46).

### Conflict of interest statement

All the authors declare that they have no conflict of interest in this work.
